# Supplementary material for: Prelimbic cortex to ventral tegmental area projection regulates early social isolation stress-potentiated heroin seeking in mice
Source: Nat Commun. 2025 Oct 29;16:9541. doi: 10.1038/s41467-025-64585-7 (PMC12572406; doi:10.1038/s41467-025-64585-7)
Supplement: Supplementary file 1 — Supplementary Information [file 41467_2025_64585_MOESM1_ESM.pdf]

Supplementary Information

Prelimbic cortex to ventral tegmental area projection regulates early social isolation stress-potentiated heroin seeking in mice

Yunwanbin Wang[1#], Shuwen Yue[1#], Fengwei Yang[3], Lu Chen[1], Archana Singh[1], Magnus Marciniak[1], Wei Wei[1], Zi-Jun Wang[1,2]

[1]Department of Pharmacology & Toxicology, School of Pharmacy, University of Kansas, Lawrence, KS, USA

[2]Cofrin Logan Center for Addiction Research and Treatment, University of Kansas, Lawrence, KS, USA

[3]Department of Biostatistics and Data Science, University of Kansas Medical Center, Kansas City, KS, USA.

[#]These two authors contribute equally to this project

[*]Corresponding author. Department of Pharmacology and Toxicology, School of Pharmacy, University of Kansas, 1251 Wescoe Hall Drive, Lawrence, KS 66045, USA. E-mail address: zjwang@ku.edu (Z.-J. Wang).

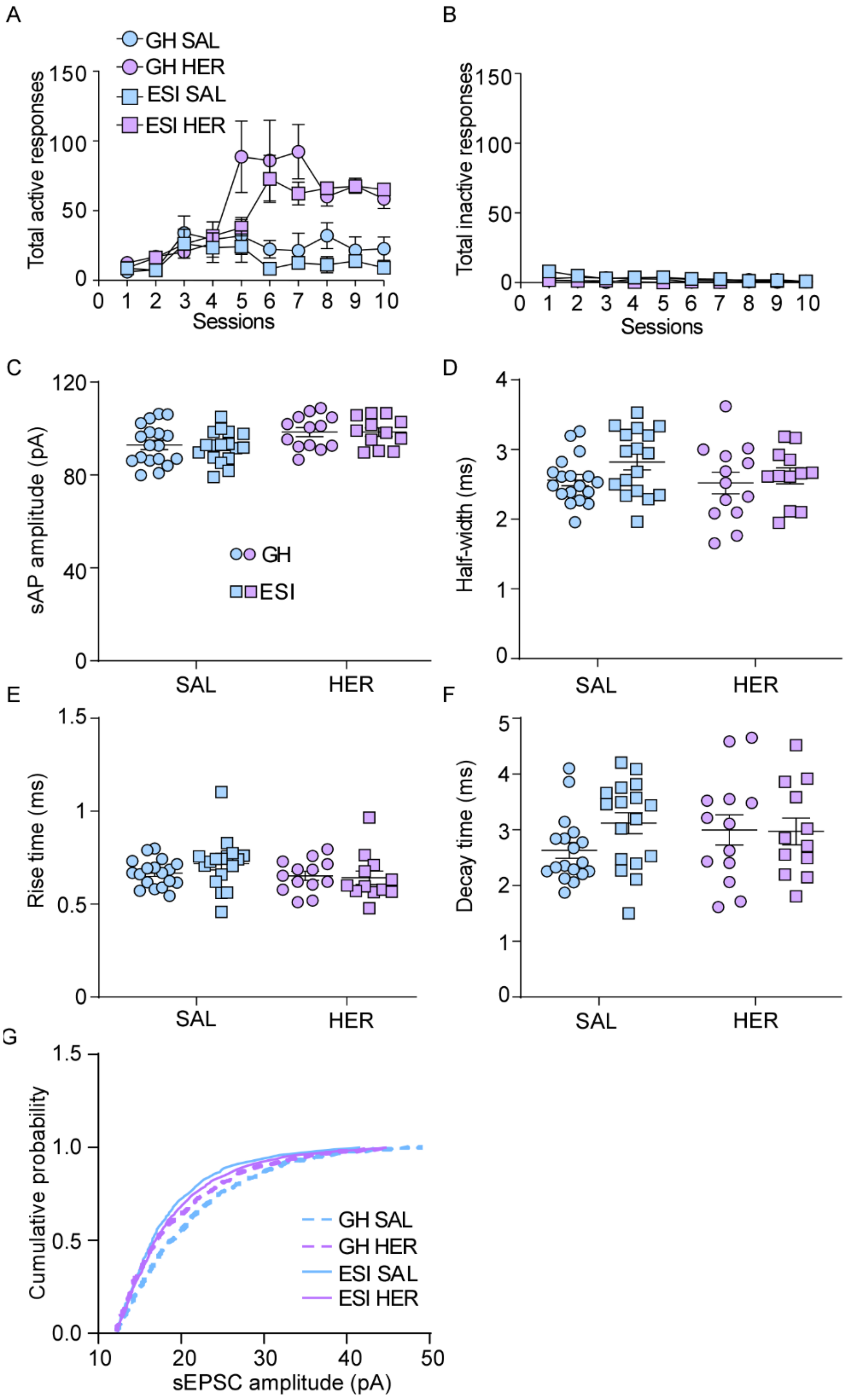

Supplementary Fig. 1 (**A-B**) Mean numbers of total active responses (A) and total inactive responses (B) per session during heroin self-administration training (0.05 mg/kg/infusion) for all groups, as shown in Fig. 1 (n = 8 mice/group). Multi-way ANOVA. (**C-F**) Bar graphs showing sAP amplitude (C), half-width (D), rise time (E), decay time (F), and cumulative probability distribution of sEPSC amplitude (G) in PrL->VTA projection (n = 18 [SAL GH], 17 [SAL ESI], 13 [HER GH], and 12 [HER ESI] cells/group). Two-way ANOVA. Data are presented as mean ± SEM.

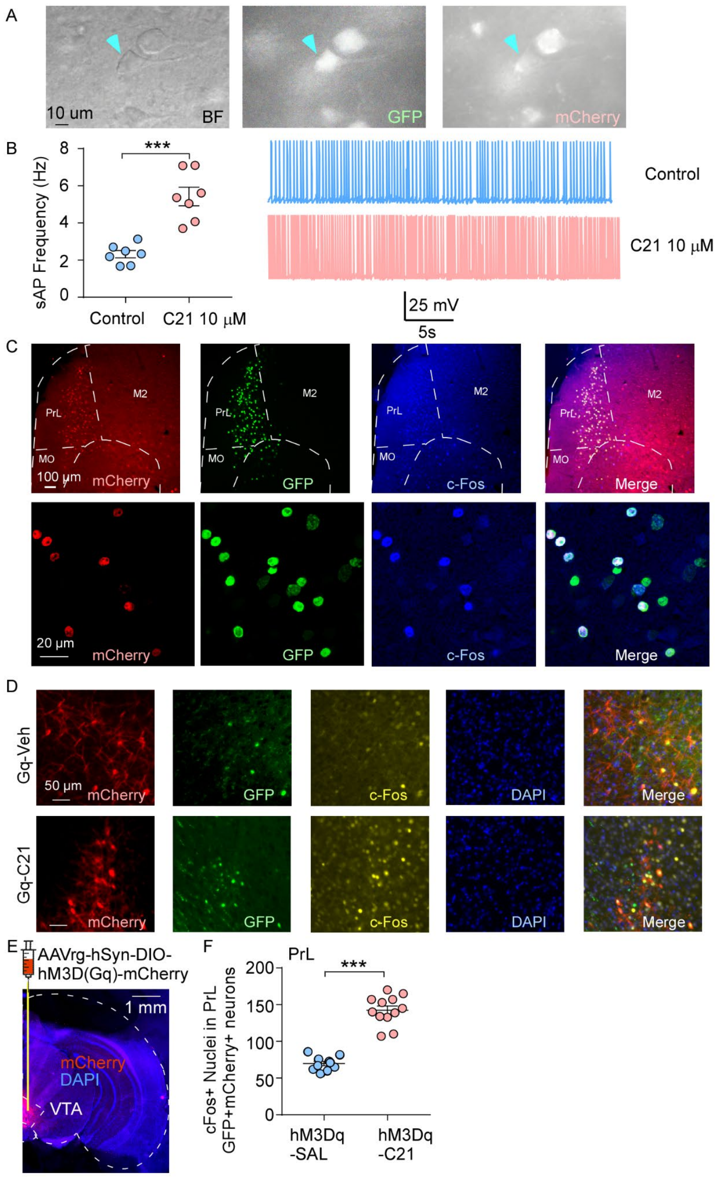

Supplementary Fig. 2 Gq-DREADDs validation. (**A**) Visualization of PrL->VTA projection in the PrL during C21 bath application recording. (**B**) Left: Bar graphs showing firing frequency of spontaneous action potential (sAP) of PrL-VTA projection before (control) and after 10 mM C21 bath application from WT mice (paired t-test, n = 7 cells, *p* = 0.0009). Right: representative traces of sAP. (**C**) Representative images showing c-Fos expression (blue) in PrL->VTA projection infected with DIO-hM3Dq-mCherry-AAVrg (red) and GFP-Cre (green) in PrL. (**D**) Representative images showing c-Fos (yellow) expression in PrL->VTA projection in PrL from vehicle (Veh) injected mice (up) and C21 injected mice (low). (**E**) Image showing the location and expression of the stereotaxically-injected retrograde DIO-DREADD-mCherry-AAV in VTA. (**F**) Number of c-Fos positive cells in the PrL->VTA projection after Veh or C21 injection (unpaired two-tailed *t*-test, n = 10 [hM3Dq-SAL] images from 3 mice/group, 12 [hM3Dq-C21] images from 4 mice/group, *p* < 0.0001). The experiment was repeated 3 times. Data are expressed as mean ± SEM, *** p < 0.001.

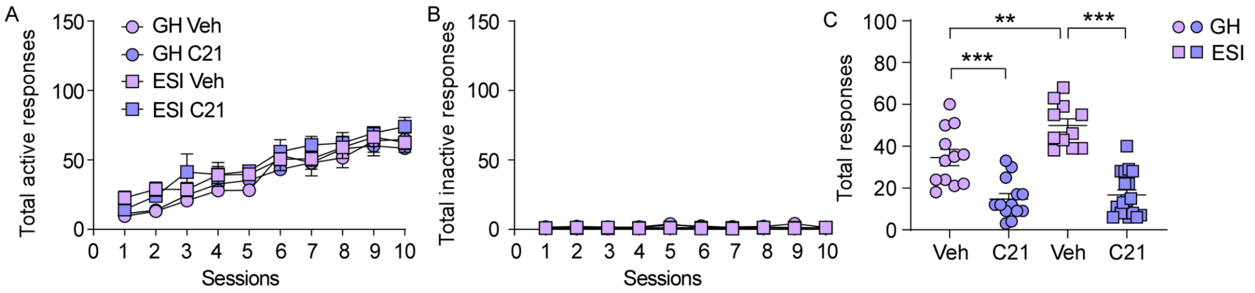

Supplementary Fig. 3 (**A-B**) Mean numbers of total active responses (A) and total inactive responses (B) per session during heroin self-administration training (0.05 mg/kg/infusion) for mice shown in Fig. 2. Multi-way ANOVA, n = 15 [GH Veh], 16 [GH C21], 14 [ESI Veh], and 21 [ESI C21] mice/group. (**C**) Mean numbers of total responses during the heroin-seeking test in Fig. 2E. Two-way ANOVA, n = 12 [GH Veh], 13 [GH C21], 11 [ESI Veh], and 18 [ESI C21] mice/group, two-way ANOVA, $F_{1, 50\ (stress)} = 7.925$, $p = 0.007$, $F_{1, 50\ (treatment)} = 75.85$, $p < 0.0001$, $F_{1, 50\ (intercept)} = 4.889$, $p = 0.0316$. Data are expressed as mean ± SEM. **p < 0.01, ***p < 0.001.

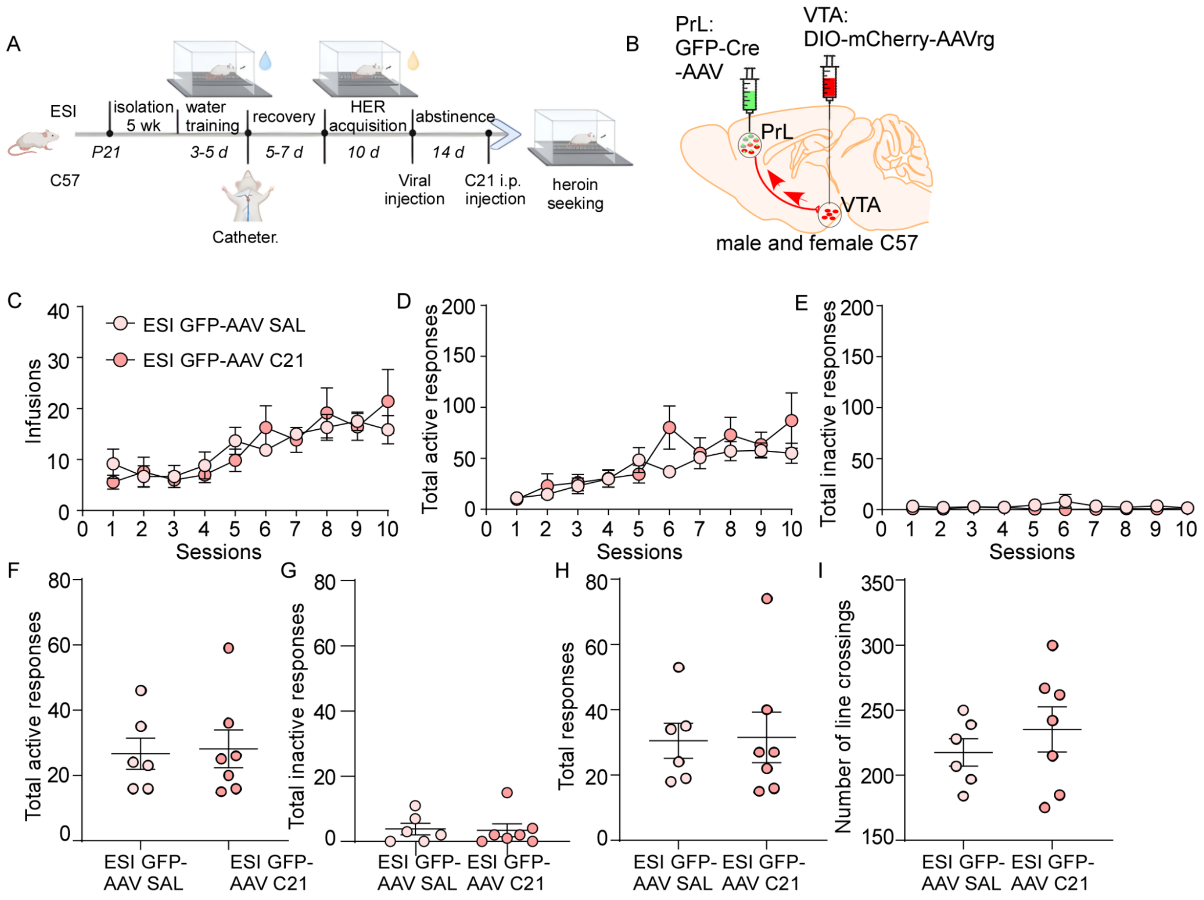

Supplementary Fig. 4 Validation of DREADDs non-specific binding behavior in ESI mice. (**A**) Experimental timeline for heroin self-administration, virus injection, C21 intraperitoneal (i.p.) injection (1 mg/kg), and heroin-seeking behavior. (**B**) Schematic representation of the injection site in WT mice. (**C-E**) Mean numbers of infusions (C), total active responses (D), and total inactive responses (E) per session during heroin self-administration training for mice injected with retrograde DIO-mCherry in VTA and GFP-AAV in PrL. n = 6 [ESI SAL] and n = 7 [ESL C21] mice/group. (**F-I**) Mean numbers of total active responses (F), total inactive responses (G), and total responses (H) during the heroin-seeking test. Locomotion (I) was tested after the heroin-seeking test. Unpaired t-test, n = 6 [ESI SAL] and n = 7 [ESL C21] mice/group. Data are expressed as mean ± SEM. **A** created in BioRender. Wang, Z. (2025) https://BioRender.com/6kfkeg5.

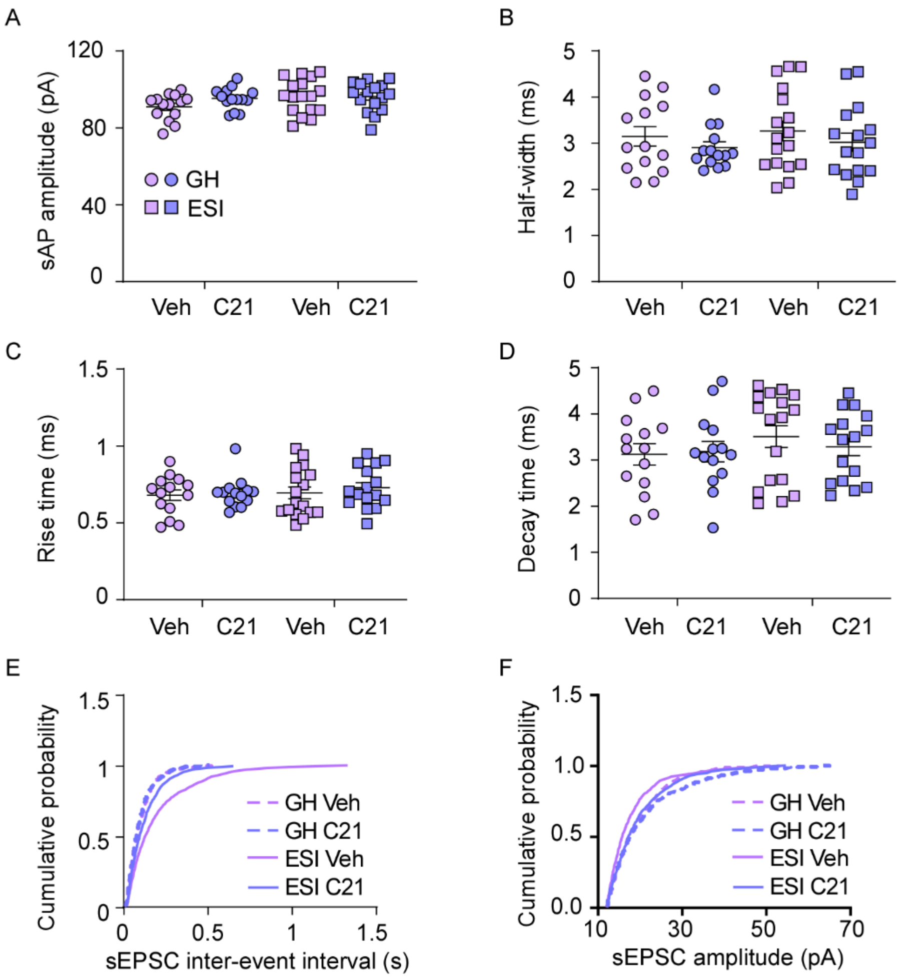

Supplementary Fig. 5 (**A-D**) Bar graphs showing sAP amplitude (A), half-width (B), rise time (C) and decay time (D) in PrL->VTA projection from mice underwent heroin self-administration and received hM3Gq AAV expression during abstinence in Fig. 2. (Two-way ANOVA, n = 14 [GH Veh], 18 [GH C21], 17 [ESI Veh], and 16 [ESI C21] cells/group). (**E-F**) Cumulative probability distributions of sEPSC frequency (E) and amplitude (F) in all four groups of mice after heroin abstinence in Fig. 2I-J. Two-way ANOVA. Data are shown as the mean ± SEM.

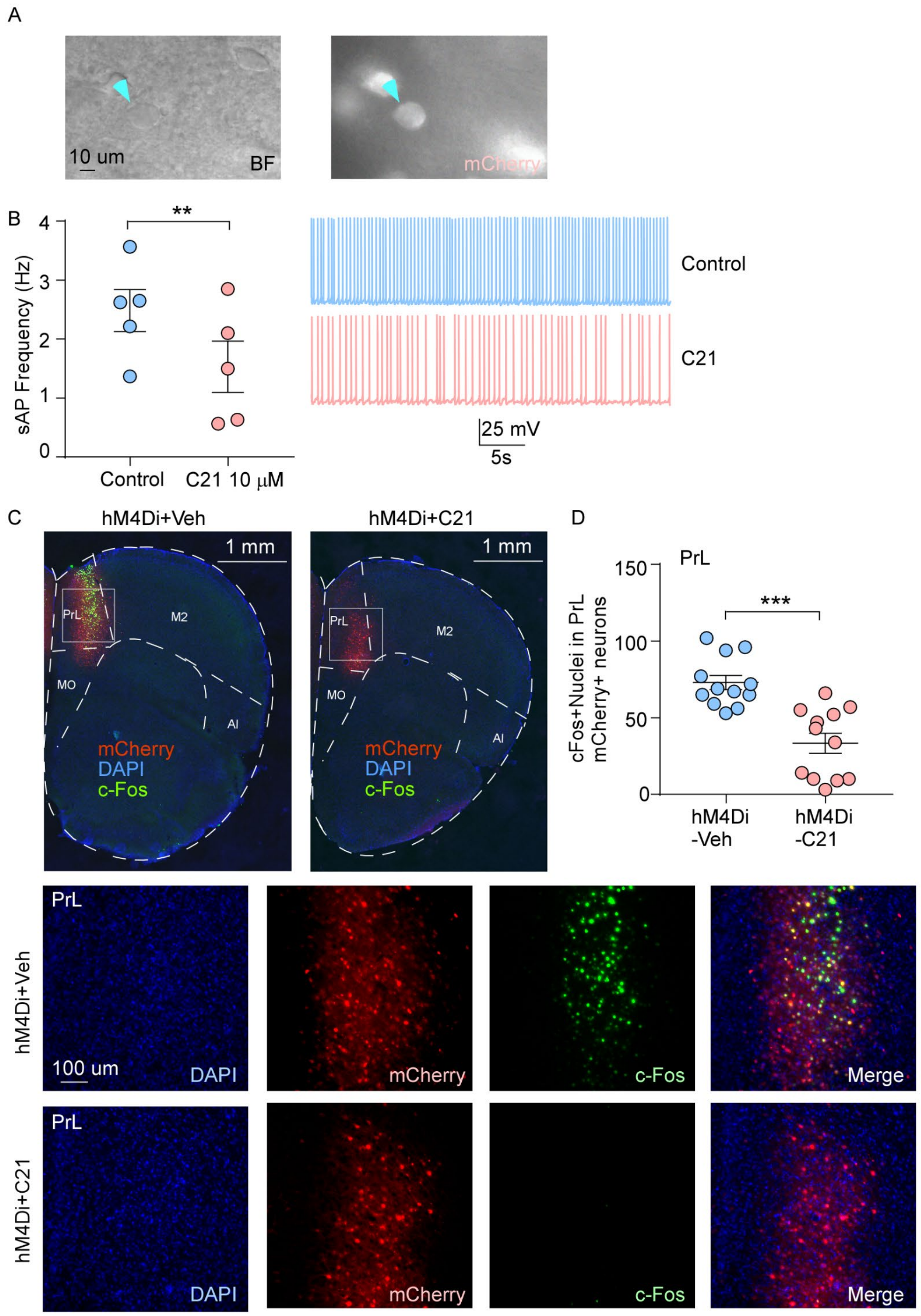

Supplementary Fig. 6 Gi-DREADDs validation for Fig. 3. (**A**) Visualization of PrL->VTA projection in the PrL during C21 bath application recording. (**B**) Left: Bar graphs showing firing frequency of spontaneous action potential (sAP) of PrL->VTA projection before (control) and after 10 mM C21 bath application from WT mice (paired t-test, n = 5 cells, $p = 0.0064$). Right: representative traces of sAP. (**C**) Representative images showing c-Fos expression (green) in PrL->VTA projection in PrL infected with DIO-hM4Di-mCherry-AAV (red) and Cre-AAVrg from Veh or C21-injected WT mice. (**D**) Number of c-Fos positive cells in the PrL->VTA projection after Veh or C21 injection (unpaired t-test, n = 12 [hM4Di-Veh] images from 3 mice/group, 12 [hM4Di-C21] images from 4 mice/group, $p < 0.0001$). The experiment was repeated 3 times. Data are shown as the mean ± SEM, ** $p < 0.01$, ***$p < 0.001$.

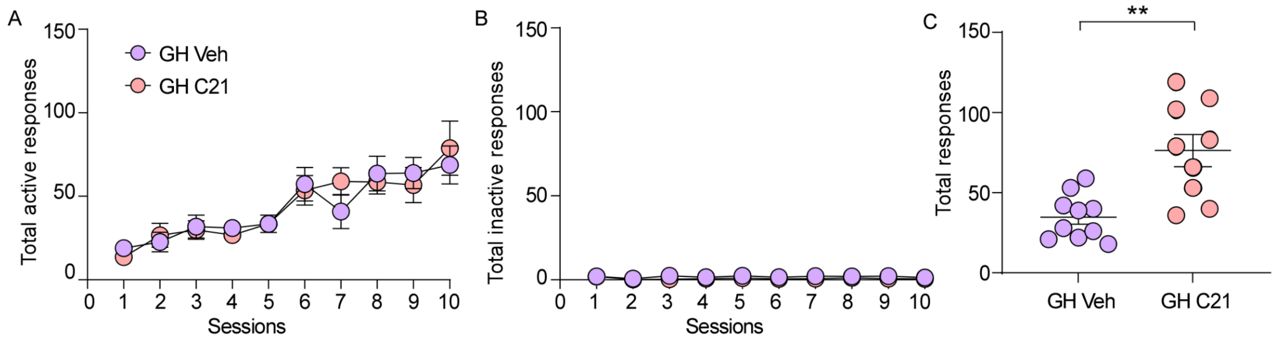

Supplementary Fig. 7 (**A-B**) Mean numbers of total active responses (A) and total inactive responses (B) per session during heroin self-administration training for all groups shown in Fig. 3. Two-way ANOVA, n = 14 [GH Veh] and 13 [GH C21] mice/group. (**C**) Mean numbers of total responses during the heroin-seeking test in Fig. 3C-F. Unpaired two-tailed *t*-test, n = 10 [GH Veh] and 9 [GH C21] mice/group, $p = 0.0011$. Data are expressed as mean ± SEM, $^{**}p < 0.01$.

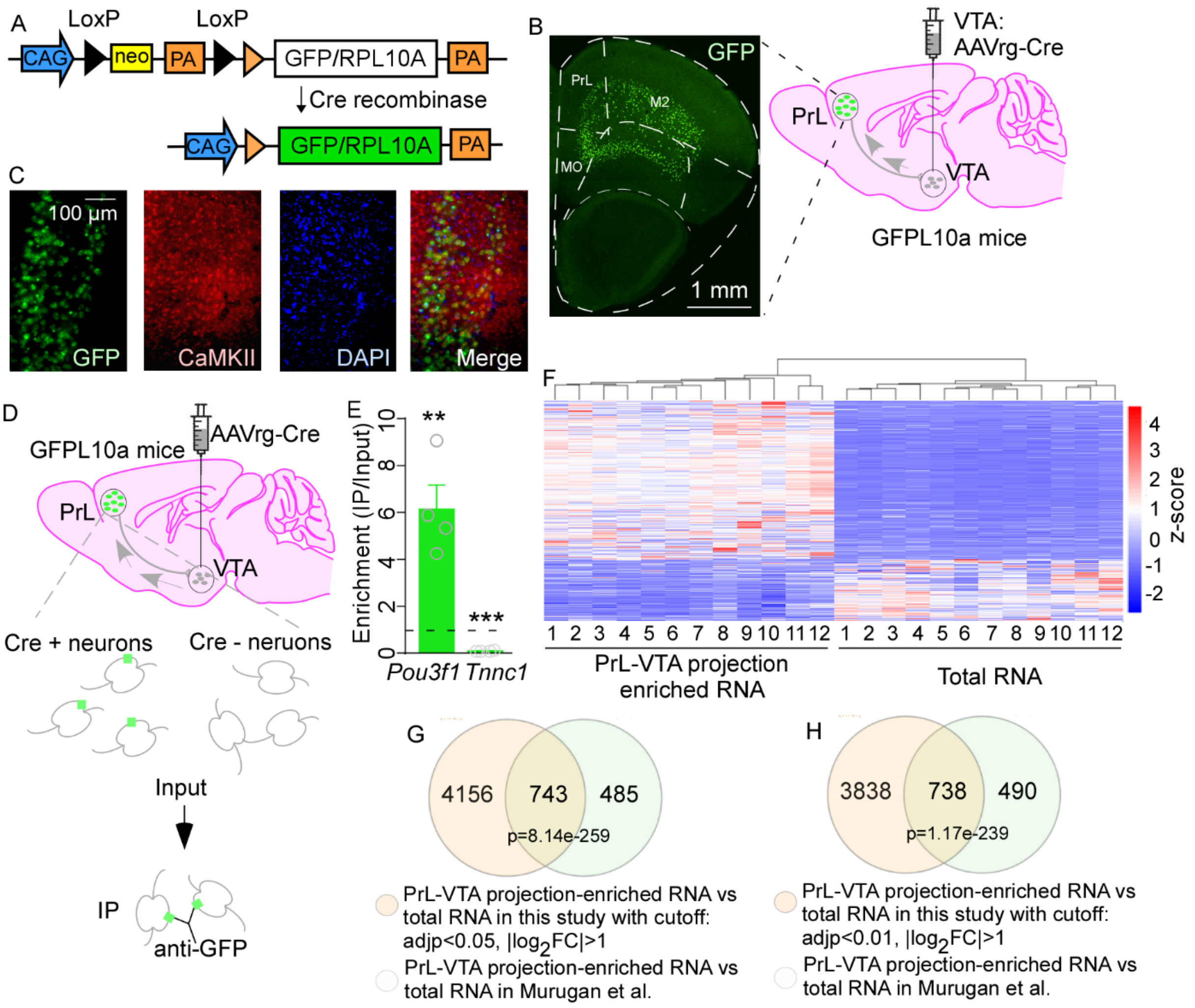

Supplementary Fig. 8 Validation of a Cre-activated TRAP allele. (**A**) Schematic of cre-dependent GFP-L10a allele and its recombined products with Cre presence. (**B**) Schematic of virus injection site in GFP-L10a$^{f/f}$ mice and GFP expression in the PrL->VTA projecting neurons. (**C**) Visualization of co-localization of CaMKII (red), GFP-L10a (green), and DAPI (blue) expression in the PrL of GFP-L10a$^{f/f}$ mice. (**D**) Schematic of translating ribosome affinity purification (TRAP) assay using GFP antibody pull-down. (**E**) Bar graph showing the fold change (relative to input [total mRNA]) in mRNA levels of marker genes for different projections in AAVrg-Cre virus-mediated TRAP mRNA from the PrL of GFP-L10a$^{f/f}$ mice (n = 4 mice/group). PrL->VTA enriched gene: *Pou3f1* (unpaired two-tailed *t*-test, *p* = 0.0026); PrL->Amygdala or PrL->Nucleus Accumbens enriched gene: *Tnnc1* (unpaired t-test, *p* < 0.0001). Data are expressed as mean ± SEM, ** p < 0.01, *** p < 0.001. (**F**) Heatmap showing the differentially expressed genes (DEGs) between PrL->VTA immunoprecipitation and total RNA. DEG cutoff: adjp<0.05, |log2FC|>1. (**G-H**) The overlap between the PrL->VTA projection enriched genes from this study and from publication (Murugan et al., 2017). G: using the cutoff of adjp<0.05, |log2FC|>1 for this study. H: using the cutoff of adjp<0.01, |log2FC|>1 for this study.

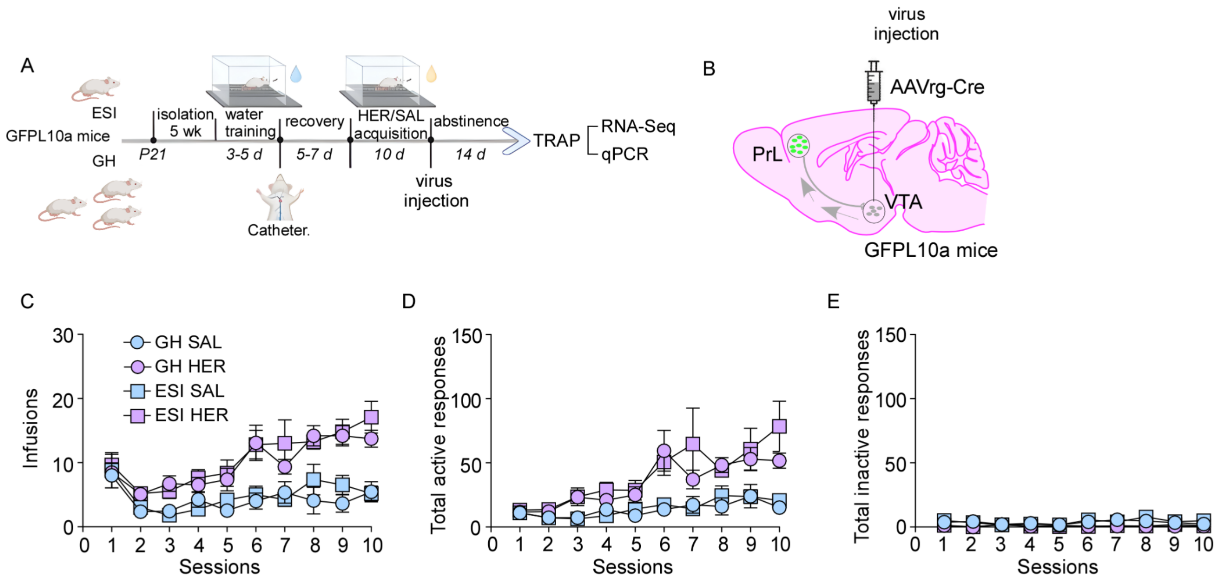

Supplementary Fig. 9 (**A**) Experimental timeline for heroin (HER) self-administration, virus injection, and RNA-Seq or qPCR analysis shown in Fig. 4. (**B**) Schematic of virus injection site in GFP-L10a$^{f/f}$ mice. (**C-E**) Mean numbers of infusions (C), total active responses (D), and total inactive responses (E) per session during heroin self-administration training for GFP-L10a$^{f/f}$ mice, as shown in Fig. 4 and Fig. 6 (n = 11 mice/group; n = 3 mice/group for RNA-Seq; n = 8 mice/group for qPCR). Multi-way ANOVA. Data are presented as mean ± SEM. **A** created in BioRender. Wang, Z. (2025) https://BioRender.com/6kfkeg5.

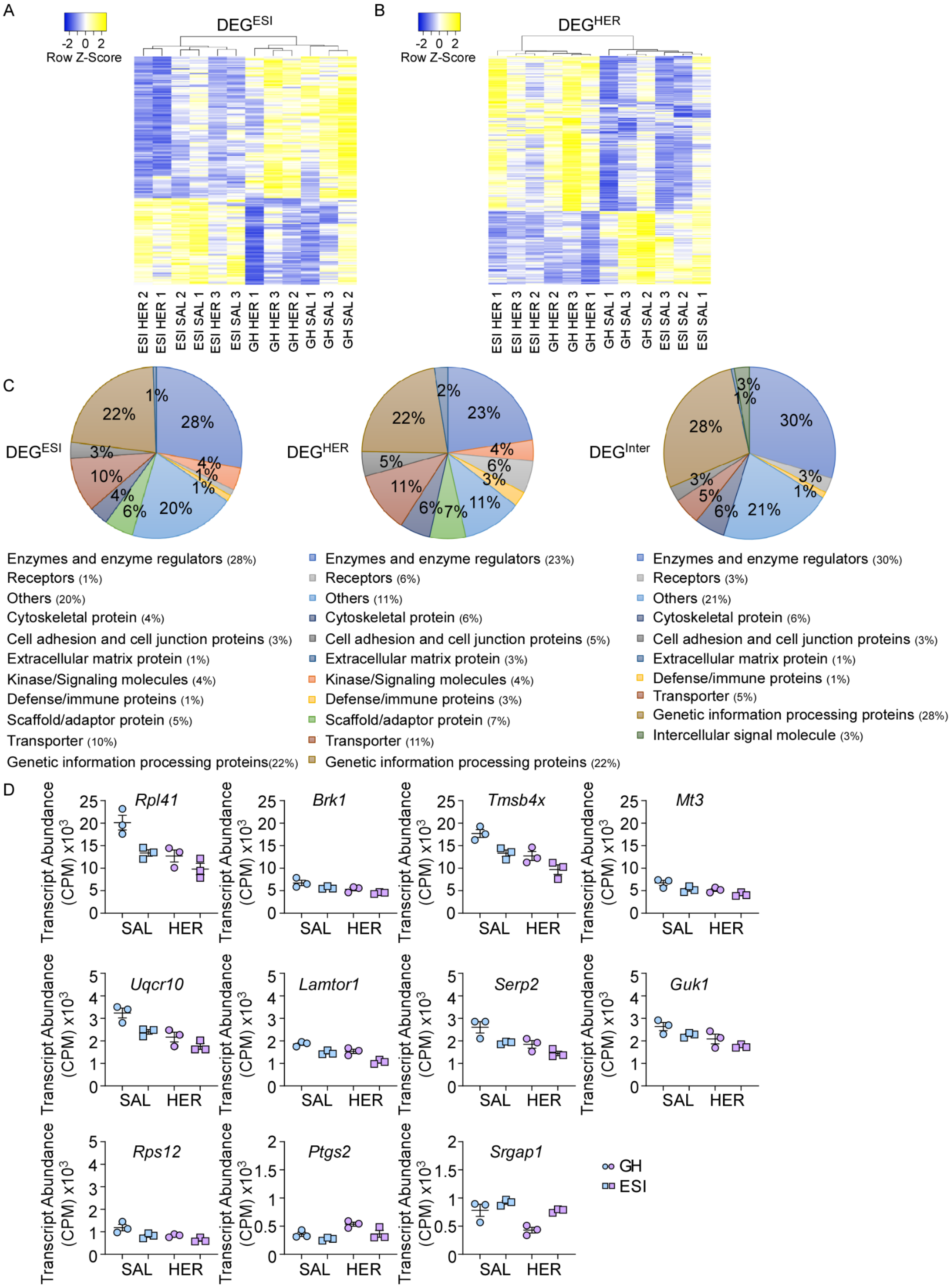

Supplementary Fig. 10 (**A-B**) Representative diagram of up-regulated (yellow) and down-regulated (blue) genes caused by ESI stress (A) and heroin (B) after heroin abstinence in PrL->VTA projection. (**C**) Functional classification of differentially expressed genes (DEGs) using the PANTHER database. Pie charts representing PANTHER protein classes in DEGs caused by ESI stress (left), heroin abstinence (middle), and stress & drug interaction (right). (**D**) Mean counts per million (CPM) reads of the overlapping DEGs caused by stress and heroin as shown in Fig. 4I (n = 3 mice/group).

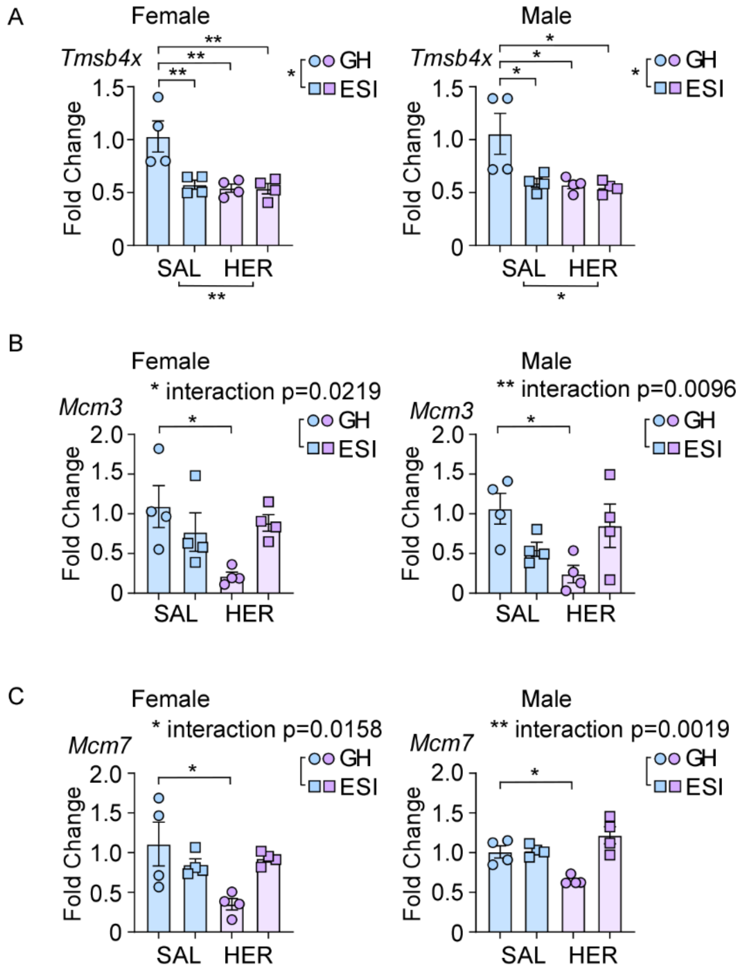

Supplementary Fig. 11 (**A-C**) qPCR results for *Tmsb4x* (A, male: $F_{1,\ 12\ \mathrm{(stress)}} = 5.75$, $p = 0.0336$, $F_{1,\ 12\ \mathrm{(drug)}} = 6.738$, $p = 0.0234$, $F_{1,\ 12\ \mathrm{(intercept)}} = 4.668$, $p = 0.0517$. Female: $F_{1,\ 12\ \mathrm{(stress)}} = 7.845$, $p = 0.016$, $F_{1,\ 12\ \mathrm{(drug)}} = 10.18$, $p = 0.0078$, $F_{1,\ 12\ \mathrm{(intercept)}} = 7.45$, $p = 0.0183$), *Mcm3* (B, male: $F_{1,\ 12\ \mathrm{(stress)}} = 0.072$, $p = 0.7936$, $F_{1,\ 12\ \mathrm{(drug)}} = 2.098$, $p = 0.1732$, $F_{1,\ 12\ \mathrm{(intercept)}} = 9.475$, $p = 0.0096$. Female: $F_{1,\ 12\ \mathrm{(stress)}} = 0.8577$, $p = 0.3726$, $F_{1,\ 12\ \mathrm{(drug)}} = 4.08$, $p = 0.0663$, $F_{1,\ 12\ \mathrm{(intercept)}} = 6.93$, $p = 0.0219$) and *Mcm7* (C, male: $F_{1,\ 12\ \mathrm{(stress)}} = 17.56$, $p = 0.0013$, $F_{1,\ 12\ \mathrm{(drug)}} = 1.414$, $p = 0.2573$, $F_{1,\ 12\ \mathrm{(intercept)}} = 15.76$, $p = 0.0019$. Female: $F_{1,\ 12\ \mathrm{(stress)}} = 1.111$, $p = 0.3126$, $F_{1,\ 12\ \mathrm{(drug)}} = 5.219$, $p = 0.0413$, $F_{1,\ 12\ \mathrm{(intercept)}} = 7.886$, $p = 0.0158$) in GH SAL, GH HER, ESI SAL and ESI HER shown in Fig. 4K and Fig. 6F, two-way ANOVA, n = 4 mice/group. Data are expressed as mean ± SEM, * $p < 0.05$, ** $p < 0.01$.

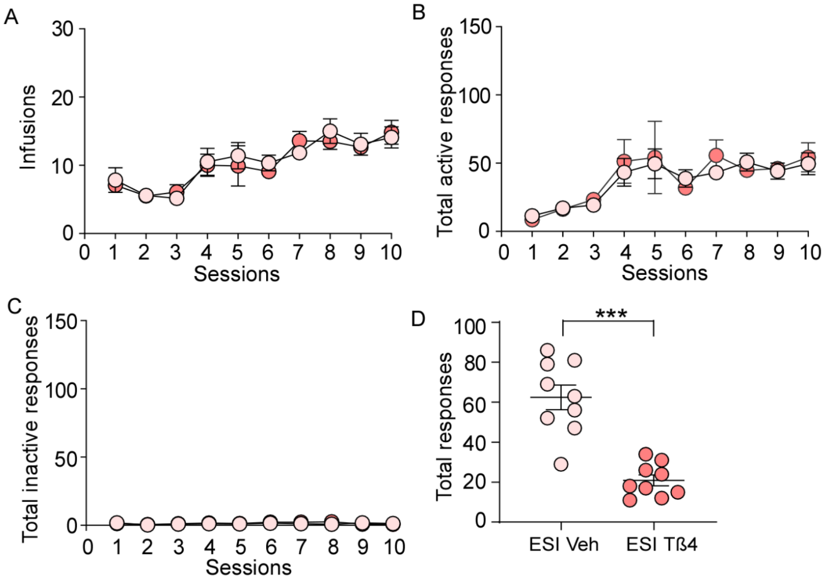

Supplementary Fig. 12 (**A-C**) Mean numbers of infusions (A), total active responses (B) and total inactive responses (C) per session during heroin self-administration training for all groups, as shown in Fig. 5. Two-way ANOVA, n = 12 mice/group. (**D**) Mean numbers of total responses (D) during heroin-seeking test, as shown in Fig. 7C-E, unpaired two-tailed *t*-test, n = 9 mice/group, $p < 0.0001$. Data are shown as the mean ± SEM. *** $p < 0.001$.

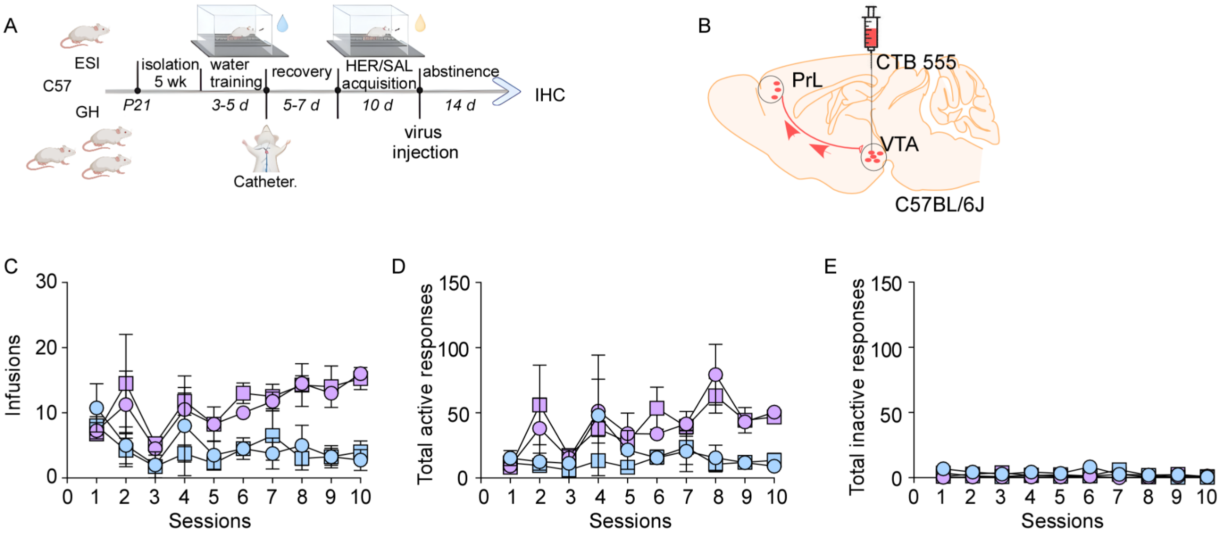

Supplementary Fig. 13 (**A**) Experimental timeline for heroin self-administration, CTB 555 injection, and immunohistochemistry (IHC) as shown in Fig. 6. (**B**) Schematic of CTB 555 injection site in WT mice. (**C-E**) Mean numbers of infusions (C), total active responses (D), and total inactive responses (E) per session during heroin self-administration training for WT mice, as shown in Fig. 6I (for IHC, n = 4 mice/group). Multi-way ANOVA. Data are presented as mean ± SEM. **A** created in BioRender. Wang, Z. (2025) https://BioRender.com/6kfkeg5.

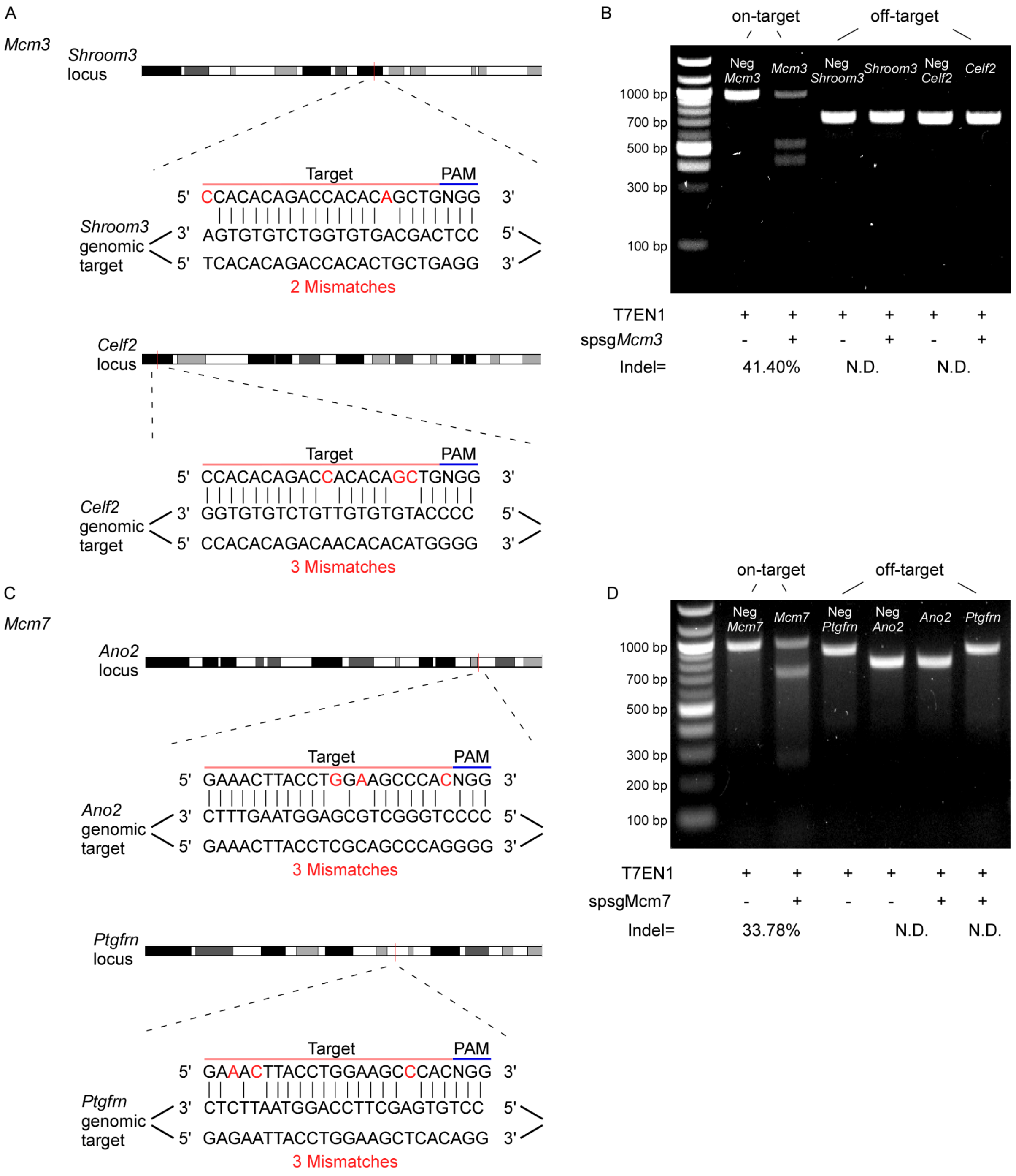

Supplementary Fig. 14 Analysis of the off-target effects of CRISPR-mediated genome editing. Related to Figure 7. (**A**) Predicted off-target site of *spsgMcm3* on *Shroom3* and *Celf2* exons. (**B**) On-target and off-target indel detection showed that *spsgMcm3* effectively induces mutations in the *Mcm3* (41.40%), but not in the *Shroom3* and *Celf2* targets. (**C**) Predicted off-target site of *spsgMcm7* on *Ano2* and *Ptgfrn* exons. (**D**) On-target and off-target indel detection showed that *spsgMcm7* effectively induces mutations in the *Mcm7* (33.78%), but not in the *Ano2* and *Ptgfrn* targets. Experiments were repeated three times.

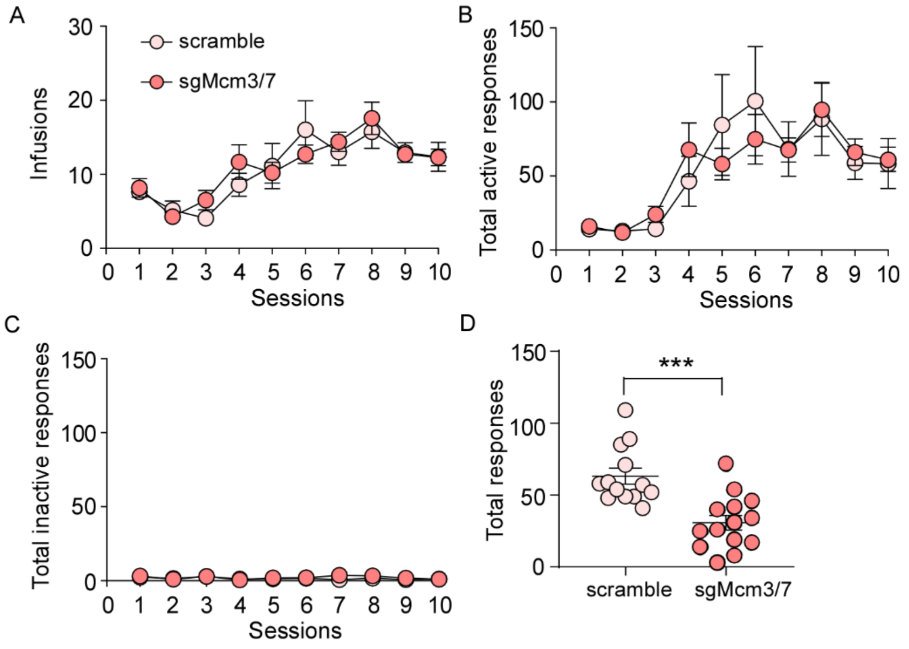

Supplementary Fig. 15 (**A-C**) Mean numbers of infusions (A) total active responses (B) and total inactive responses (C) per session during heroin self-administration training for all groups shown in Fig. 7 G-K. Two-way ANOVA, n = 19 [scramble] and 18 [sgMcm3/7] mice/group. (**D**) Mean numbers of total responses during heroin-seeking test in Fig. 7 G-H. Unpaired two-tailed *t*-test, n = 13 [scramble] and 14 [sgMcm3/7] mice/group, $p = 0.0002$. Data are shown as the mean ± SEM. *** $p < 0.001$.

Supplementary Table 1: Sequences of primers used for qPCR validation experiment

Gene name	Forward Primer 5'- 3'	Reverse Primer 5'- 3'	Product length (bp)
Gapdh	GACAACAGCCTCAAGATCATCAG	ATGGCATGGACTGTGGTCATGAG	122
Rpl41	GACACCGAGCACGCCATTAAA	TCTTCTTCCGCCACTTCGCT	110
Uqcr10	CACATCAACGAGGGGAAACT	ACGGCAACTTGAAACTCATCC	154
Brk1	GCGAACCGGGAGTACATTGAG	AGTCTCACCCTTTGTCACCCTC	171
Tmsb4x	ATCCTCTGCCTTCAAAAGAAACAAT	AAGGGGCAGCACAGTCATTT	185
Srgap1	ATGGGGATTTGGAGACATTCGT	AGGGTTCTCACCTCTCTCG	176
Mt3	CCCTGTCCTACTGGTGGTTC	CACACTTCTCACATCCGGCA	118
Serp2	CCATCGACTCTCGCAAGCTAT	AAGAGCAGAGTCCTTTCCGC	270
Lamtor1	GCCAAGACAGCTAGCAACATC	ACGCGGATCTGAGAAAGTGC	269
Ptgs2	TGGGGGAAGAAATGTGCCAA	CAGCCATTTCCTTCTCTCCTGT	161
Mcm3	TTCCGTGGCGGGAAAACTTG	AAATGCCTTGGTCCTCCTCGT	186
Mcm7	GCGTTCGTTTTCTGCTTCCC	CGATGAGCCAGATGAACCAACT	200
Pou3f1	TTCAAGCAACGACGCATCAAG	CCGCGATCTTGTCCAGGTTG	220
Tnnc1	CAGTAGCCTGTCCTGTGAGC	GCCACTGCCATCCTCGTCTA	240

Supplementary Table 2: MetaData for TRAP-sequencing data analysis using DEseq2

id	dex	drug
ESI_HER_1	ESI	HER
ESI_HER_2	ESI	HER
ESI_SAL_1	ESI	SAL
ESI_SAL_2	ESI	SAL
ESI_HER_3	ESI	HER
ESI_SAL_3	ESI	SAL
GH_SAL_1	GH	SAL
GH_HER_1	GH	HER
GH_SAL_2	GH	SAL
GH_HER_2	GH	HER
GH_HER_3	GH	HER
GH_SAL_3	GH	SAL

Supplementary Table 3: Predicted Off-target sites for gRNA targeting Mcm3 and Mcm7

		Target site	Chromosome	Position	Direction	Mismatches	DNA sequence	testing primer Forward 5'- 3'	testing primer Reverse 5'- 3'	Product length (bp)
spsg*Mcm3*	on-target	*Mcm3*	chr1	20814797	+	0	CCACACAGACCACACAGCTGAGG	TACAGGCTGTCGTTGTCGTC	AGTGAACACTGCGAACGACT	949
	off-target	*Shroom3*	chr5	92870516	+	2	tCACACAGACCACACtGCTGAGG	TGACTTTCCCGTAGGGTTGC	TGGGGACACTTAGGATGCAC	740
	off-target	*Celf2*	chr2	7118879	-	3	CCACACAGACaACACAcaTGGGG	GTTGGGGGAATCTCGGTGTT	CAACACGCATGCTCACACTC	727
spsg*Mcm7*	on-target	*Mcm7*	chr5	138168136	-	0	GAAACTTACCTGGAAGCCCACTGG	TATGAGTCCTGCAGCTGGTTA	CCTCCCTCCTAAGTCTCTTGTCC	985
	off-target	*Ano2*	chr6	125690729	-	3	GAAACTTACCTcGcAGCCCAgGGG	GCTGCACCTGAGAAAGGGAT	AATAGCCAGCAACTCCCACC	779
	off-target	*Ptgfrn*	chr3	101063580	+	3	GAgAaTTACCTGGAAGCtCACAGG	CAGCCTCAACCTGCACACTA	GGCCCACAGGAAGCTGTAAT	870
