# Supplementary material for: Prelimbic cortex to ventral tegmental area projection regulates early social isolation stress-potentiated heroin seeking in mice
Source: Nat Commun. 2025 Oct 29;16:9541. doi: 10.1038/s41467-025-64585-7 (PMC12572406; doi:10.1038/s41467-025-64585-7)
Supplement: Supplementary file 2 — Description of Additional Supplementary Files [file 41467_2025_64585_MOESM2_ESM.pdf]

Description of Additional Supplementary Files

Supplementary Data 1. Statistics details for data in the figures

Supplementary Data 2. DEGs list for PrL->VTA projection enriched RNA vs total RNA

Supplementary Data 3. DEG-ESI: DEGs list for the comparison of ESI vs GH

Supplementary Data 4. DEG-HER: DEGs list for the comparison of HER vs SAL

Supplementary Data 5. The overlapping Gene Ontology (GO) Biological Process (BP) terms identified from DEG-ESI (DEGs from the ESI vs. GH comparison) and DEG-HER (DEGs from the HER vs. SAL comparison)

Supplementary Data 6. DEG-inter: DEGs for the ESI and HER interaction effect. FC=(ESI-HER/ESI-SAL)/(GH-HER/GH-SAL)

Supplementary Data 7. String interaction score from String Enrichment analysis using the top 40 DEGs from the DEG-iner list
