# Supplementary material for: Prelimbic cortex to ventral tegmental area projection regulates early social isolation stress-potentiated heroin seeking in mice
Source: Nat Commun. 2025 Oct 29;16:9541. doi: 10.1038/s41467-025-64585-7 (PMC12572406; doi:10.1038/s41467-025-64585-7)
Supplement: Supplementary file 10 — Reporting Summary [file 41467_2025_64585_MOESM10_ESM.pdf]

Corresponding author(s): Zi-Jun Wang

Last updated by author(s): Aug 13, 2025

Reporting Summary

Nature Portfolio wishes to improve the reproducibility of the work that we publish. This form provides structure for consistency and transparency in reporting. For further information on Nature Portfolio policies, see our Editorial Policies and the Editorial Policy Checklist.

Statistics

For all statistical analyses, confirm that the following items are present in the figure legend, table legend, main text, or Methods section.

n/a	Confirmed	
☐	☒	The exact sample size (*n*) for each experimental group/condition, given as a discrete number and unit of measurement
☐	☒	A statement on whether measurements were taken from distinct samples or whether the same sample was measured repeatedly
☐	☒	The statistical test(s) used AND whether they are one- or two-sided<br>*Only common tests should be described solely by name; describe more complex techniques in the Methods section.*
☒	☐	A description of all covariates tested
☐	☒	A description of any assumptions or corrections, such as tests of normality and adjustment for multiple comparisons
☐	☒	A full description of the statistical parameters including central tendency (e.g. means) or other basic estimates (e.g. regression coefficient) AND variation (e.g. standard deviation) or associated estimates of uncertainty (e.g. confidence intervals)
☐	☒	For null hypothesis testing, the test statistic (e.g. *F*, *t*, *r*) with confidence intervals, effect sizes, degrees of freedom and *P* value noted<br>*Give P values as exact values whenever suitable.*
☒	☐	For Bayesian analysis, information on the choice of priors and Markov chain Monte Carlo settings
☒	☐	For hierarchical and complex designs, identification of the appropriate level for tests and full reporting of outcomes
☒	☐	Estimates of effect sizes (e.g. Cohen's *d*, Pearson's *r*), indicating how they were calculated

Our web collection on statistics for biologists contains articles on many of the points above.

Software and code

Policy information about availability of computer code

Data collection	Med-PC V software suite 5.15.02, Any-maze version 7.16, Clampex 11.0 data acquisition system, MultiClamp 2.2.2, NIS elements imaging software 5.41.02, NextSseq2000 platform.
Data analysis	ClampFit 11.2, miniAnalysis 6.0.7, SPSS 29.00.2(20), GraphPad Prism 9, Cytoscape_v3.10.3, R-4.4.1, R studio 1022.12.0+353

For manuscripts utilizing custom algorithms or software that are central to the research but not yet described in published literature, software must be made available to editors and reviewers. We strongly encourage code deposition in a community repository (e.g. GitHub). See the Nature Portfolio guidelines for submitting code & software for further information.

Data

Policy information about availability of data

All manuscripts must include a data availability statement. This statement should provide the following information, where applicable:

- Accession codes, unique identifiers, or web links for publicly available datasets
- A description of any restrictions on data availability
- For clinical datasets or third party data, please ensure that the statement adheres to our policy

The data supporting the findings of this study are available in the article and in its online supplementary material. Genomic data are available through GEO database (GSE293281).

Research involving human participants, their data, or biological material

Policy information about studies with human participants or human data. See also policy information about sex, gender (identity/presentation), and sexual orientation and race, ethnicity and racism.

Reporting on sex and gender	N/A
Reporting on race, ethnicity, or other socially relevant groupings	N/A
Population characteristics	N/A
Recruitment	N/A
Ethics oversight	N/A

Note that full information on the approval of the study protocol must also be provided in the manuscript.

Field-specific reporting

Please select the one below that is the best fit for your research. If you are not sure, read the appropriate sections before making your selection.

☒ Life sciences ☐ Behavioural & social sciences ☐ Ecological, evolutionary & environmental sciences

For a reference copy of the document with all sections, see nature.com/documents/nr-reporting-summary-flat.pdf

Life sciences study design

All studies must disclose on these points even when the disclosure is negative.

Sample size	We previously conducted a power analysis of the our preliminary data using G*power. The large Cohen's d effect size140, f=0.96 with 8-18 mice/group for behavior, 12-20 cells/group for electrophysiology, and 5-8 replicates/group for biochemical studies, provides a power of 0.95 for P<0.05. Based on this experience and previous publications, we decided the sample size for the current study.
Data exclusions	Data were excluded based on pre-established criteria to ensure accuracy and experimental validity. Specifically:<br>Animals were excluded if they had incorrect viral expression or cannula placement, confirmed through postmortem histological analysis;<br>Electrophysiology data were excluded if recordings showed unstable baselines, excessive noise, or poor seal quality;<br>Behavioral data were excluded if animals did not acquire heroin self-administration criteria;<br>These criteria were established prior to data collection based on standard lab practices.<br>For all samples recorded and complied for final data analysis, there were no outliers (Graph Pad Prism's Outlier test).
Replication	All of our experiments were run in different cohorts (at least 2-3 times). All replications were successful.
Randomization	Animals were randomly allocated to experimental groups to minimize litter effects and selection bias. Following heroin self-administration, animals were allocated to treatment groups based on their self-administration behavior to ensure groups were balanced for drug intake and performance. This approach was used to minimize behavioral bias across treatment conditions. Sex was balanced across groups.
Blinding	We performed all of our experiments in a blinded manner (i.e., the experimenter will not know the treatments during the tissue preparation and data analyses).

Reporting for specific materials, systems and methods

We require information from authors about some types of materials, experimental systems and methods used in many studies. Here, indicate whether each material, system or method listed is relevant to your study. If you are not sure if a list item applies to your research, read the appropriate section before selecting a response.

Materials & experimental systems

n/a	Involved in the study	
☐	☒	Antibodies
☐	☒	Eukaryotic cell lines
☒	☐	Palaeontology and archaeology
☐	☒	Animals and other organisms
☒	☐	Clinical data
☒	☐	Dual use research of concern
☒	☐	Plants

Methods

n/a	Involved in the study	
☒	☐	ChIP-seq
☒	☐	Flow cytometry
☒	☐	MRI-based neuroimaging

Antibodies

Antibodies used	c-Fos (Abcam, ab190289; Cell Signalling, #2250S), CaMKII (Abcam, ab22609), Mcm3 (Santa Cruz, sc-390480), Mcm7 (Santa Cruz, sc-9966)
Validation	All antibodies are validated for western blot and immunohistochemistry studies for mouse, and have been cited in publications.

Eukaryotic cell lines

Policy information about cell lines and Sex and Gender in Research

Cell line source(s)	N2A (mouse, ATCC, CCL-131)
Authentication	The cell line has not been authenticated.
Mycoplasma contamination	The cells tested negative for mycoplasma contamination on a routine base.
Commonly misidentified lines (See ICLAC register)	No commonly misidentified cell line was used.

Animals and other research organisms

Policy information about studies involving animals; ARRIVE guidelines recommended for reporting animal research, and Sex and Gender in Research

Laboratory animals	C57BL/6J mice (stock number 000664), B6;129S4-Gt(ROSA)26Sortm9(EGFP/Rpl10a)Amc/J (GFP-L10a) mice (stock number 024750), B6;129S6-Gt(ROSA)26Sortm2(CAG-NuTRAP)Evdr/J (NuTRAP) mice (stock number 029899), and B6J.129(Cg)-Gt(ROSA)26Sortm1.1(CAG-cas9*,-EGFP)Fezh/J,mice(Cas9flox/flox mice, stock number 026179).
Wild animals	N/A
Reporting on sex	Both male and female mice were used in this study. Part of the experiment has considered sex as an independent factor.
Field-collected samples	N/A
Ethics oversight	All animal experiments were performed under the approval of the Institutional Animal Care and Use Committee (IACUC), University of Kansas.

Note that full information on the approval of the study protocol must also be provided in the manuscript.

Plants

Seed stocks	N/A
Novel plant genotypes	N/A
Authentication	N/A
